# Evidence for the Involvement of Pleckstrin Homology Domain-Containing Proteins in the Transport of Enterocin DD14 (EntDD14); a Leaderless Two-Peptide Bacteriocin

**DOI:** 10.3390/ijms222312877

**Published:** 2021-11-28

**Authors:** Adrián Pérez-Ramos, Rabia Ladjouzi, Abdellah Benachour, Djamel Drider

**Affiliations:** 1 UMR Transfrontalière BioEcoAgro 1158, University Lille, INRAE, University Liège, UPJV, YNCREA, University Artois, University Littoral Côte d’Opale, ICV—Institut Charles Viollette, 59000 Lille, France; adrian.perez-ramos@univ-lille.fr (A.P.-R.); rabia.ladjouzi@univ-lille.fr (R.L.); 2 UR Risques Microbiens, Normandie University, UNICAEN, U2RM, 14000 Caen, France; abdellah.benachour@unicaen.Fr

**Keywords:** leaderless bacteriocins, EntDD14, pleckstrin homology domain, bacteriocin transport

## Abstract

Bacteriocins synthesis is initiated from an inactive precursor, which is composed of an N-terminal leader peptide attached to a C-terminal pro-peptide. However, leaderless bacteriocins (LLB) do not possess this N-terminal leader peptide nor undergo post-translational modifications. These atypical bacteriocins are observed to be immediately active after their translation in the cytoplasm. However, although considered to be simple, the biosynthetic pathway of LLB remains to be fully understood. Enterocin DD14 (EntDD14) is a two-peptide LLB produced by *Enterococcus faecalis* 14, which is a strain isolated from meconium. In silico analysis of DNA encoding EntDD14 located a cluster of 10 genes *ddABCDEFGHIJ*, where *ddE* and *ddF* encode the peculiar DdE and DdF proteins, carrying pleckstrin homology (PH) domains. These modules are quite common in Eucarya proteins and are known to be involved in intracellular signaling or cytoskeleton organization. To elucidate their role within the EntDD14 genetic determinants, we constructed deletion mutants of the *ddE* and *ddF* genes. As a result, the mutants were unable to export EntDD14 outside of the cytoplasm even though there was a clear expression of structural genes *ddAB* encoding EntDD14, and genes *ddHIJ* encoding an ABC transporter. Importantly, in these mutant strains (Δ*ddE* and Δ*ddF*), EntDD14 was detected by mass spectrometry in the intracellular soluble fraction exerting, upon its accumulation, a toxic effect on the producing strain as revealed by cell-counting and confocal microscopy analysis. Taken together, these results clearly indicate that PH domain-containing proteins, such as DdE and DdF, are involved in the transport of the leaderless two-peptide EntDD14.

## 1. Introduction

Bacteriocins are produced by a wide range of Gram-negative bacteria (GNB), Gram-positive bacteria (GPB), and Archaea [1,2,3], permitting them to compete with congeners and thrive in their ecological niches. Of note, bacteriocins produced by GNB (named microcins), and those produced by GPB have been intensively studied and have shown similarities in their biosynthetic pathways and differences in their modes of action [4,5].

Due to the increasing number of bacteriocins discovered, their classification is regularly revised and updated. The most recent revision proposes the inclusion of bacteriocins from both GPB and GNB into two main classes [6]. Briefly, class I contains bacteriocins with a molecular weight of less than 5 kDa and undergoing post-translational modifications (PTMs), while class II comprises principally unmodified bacteriocins of 6–10 kDa, including or not stabilizing disulfide bridges [6].

Bacteriocins are known to be synthesized as inactive pre-peptides that undergo a maturation process, proceeding to fully active peptides. Notably, the precursor peptide carries an N-terminal leader region and C-terminal core region, which is subjected to enzymatic PTMs, leading therefore to ribosomally synthesized and post-translationally modified peptides (RiPPs) [7]. The leader peptide is often cut off at a double-glycine proteolytic processing site during export by an ATP-binding cassette (ABC) transporter, and the mature peptide is released into the external environment. The cleavage of the leader peptide can be performed by the same ABC transporter (SunT-type transporter) or by an associated protein (NisT-type) [5,8]. However, other bacteriocins, such as enterocin P, contain an N-terminal *sec*-dependent sequence leader and follow another secretory pathway, being secreted by general sec-dependent export systems [9]. Furthermore, there are bacteriocins with leader peptide whose transport systems remain to be determined [8].

The export of bacteriocins is tightly associated with a complex immunity machinery, since the bacteriocin-producing bacteria need to protect themselves from the toxicity of their own bacteriocins. This immunity can be exerted by dedicated proteins and/or ABC transporters [10]. This is the case of NisEFG for nisin [11] or As-48EFGH for the cyclic enterocin AS-48 [12].

Cintas et al. [13] reported that enterocin L50, previously classified as a pediocin-like bacteriocin, was actually composed of two peptides, L50A/B, which was transported without any processing, leading to the emergence of the leaderless bacteriocins (LLB) group. These atypical bacteriocins do not undergo any PTM or processing and are thought to be active immediately after their translation [14]. Although LLB have no leader peptide for their export, they are also transported by ABC transporters. For instance, aureocin A70 is thought to be externalized by a single-component ABC transporter AurT [15]. Whereas a three-component ABC transporter is involved in both transport and immunity to LLB aureocin A53 [16], this transporter is not the only pathway, because when removed, there is still bacteriocin externalization. Therefore, the uncharacterized Orf8 protein has been reported to be essential for LLB aureocin A53 transport [16].

EntDD14 is a two-peptide LLB produced by *Enterococcus faecalis* 14 isolated from meconium [17,18], and its activity is essentially directed against GPB including *Clostridium perfringens*, *Listeria*, and other enterococcal strains [18]. The two peptides of EntDD14 (EntDD14A/B) are 100% identical to those of MR10 [19] and Ent7 [20] enterocins and 98% and 95% identical to those of L50A/B [13], respectively. Recent studies on EntDD14 showed that deletion of the ATP-binding protein component of the putative ABC transporter reduced the bacteriocin externalization by about 75%, and this ABC transporter is not involved in any bacterial self-immunity system to our current knowledge [21].

In silico analysis of the EntDD14 operon has revealed two proteins, named DdE and DdF, which carry pleckstrin homology (PH) domains. Of note, the *ddE* and *ddF* genes are located upstream of those encoding the ABC transporter *ddHIJ*. The PH domain is abundant in Eukarya, where it is associated with diverse functions, such as intracellular signaling and cytoskeletal organization [22,23]. The presence of the PH domain module in bacterial proteins was discovered by Xu et al. [24], who reported the bacterial PH domains, PH*b*1 and PH*b*2. Interestingly, the in silico analysis undertaken for this work showed that the DdE and DdF proteins may contain one or more PH*b*2 domains. Remarkably, the deletion of *ddE* or *ddF* genes encoding the DdE or DdF protein, which carry the PH domain, led to a very interesting result; namely, following the deletion of *ddE* or *ddF*, the two-peptide LLB EntDD14 was no longer externalized, and its intracellular accumulation exerted a toxic effect on the bacteriocin-producing bacteria.

## 2. Results

### 2.1. In Silico Characterization of DdE and DdF Proteins

**Figure 1:**
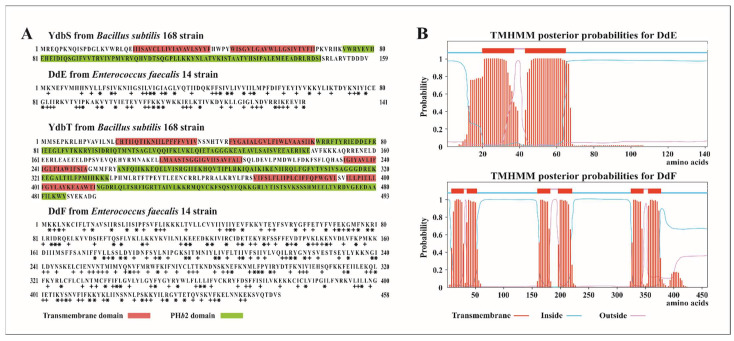
(**A**) Amino acid sequences of the YdbS and YdbT proteins, where their transmembrane and PH*b*2 domains are highlighted (data taken from PDB). In addition, amino acids sequences of DdE and DdF proteins are depicted showing their identical (*) and positive (+) amino acid residues when aligned with YdbS and YdbT, respectively. (**B**) Transmembrane domains predicted for the DdE and DdF proteins.

The EntDD14 cluster is composed of 10 genes: *ddABCDEFGHIJ*. Genes *ddAB* encode the two peptides of EntDD14, while *ddHIJ* encode the ABC transporter [21]. The other remaining five genes, *ddCDEFG*, were allocated to proteins of unknown functions. Of note, genes *ddEF*, which encode two proteins of 141 (DdE) and 458 (DdF) amino acid residues, displayed homology with proteins carrying PH domains. The BLAST tool analysis revealed the presence of the conserved protein domain family YdbT (Genbank accession COG3428), within the primary structure of the DdF protein. Remarkably, this domain is based on the uncharacterized membrane protein YdbT, which contains the PH*b*2 (bacterial pleckstrin homology) domain that was first described in *Bacillus subtilis*. In the genome of *B. subtilis* ssp. *subtilis* 168 strain, the protein YdbT of 493 amino acid residues is associated to heterologous antibiotic resistance (Genbank accession NP_388341.1), although no experimental evidence supported the allocation of such a function. Upstream of YdbT, we detected another PH domain containing the protein named YdbS, of 159 amino acids, which has the conserved protein domain family YdbS (Genbank accession COG3402). The reported data from the Protein Data Bank (PDB) revealed that YdbS protein has two transmembrane (TM) domains and one PH*b*2 domain, while the YdbT protein has six TM domains and three PH*b*2 domains (Figure 1A).

Of note, genes encoding homologous proteins of YdbT and YdbS were detected in the genomes of bacteria such as *Staphylococcus aureus*, *Corynebacterium glutamicum*, or *L. innocua* (Table 1). In all these species, the two genes encoding YdbS- and YdbT-like proteins are contained in a same locus exhibiting a common transcriptional orientation, as is the case of *ddE* and *ddF* genes. Furthermore, DdE and DdF proteins share sequence homology with *B. subtilis* YdbS and YdbT, respectively, although they are only 17% and 18% identical (Table 1 and Figure 1A).

According to BLAST analyses in the *E. faecium* L50 strain, which produces the leaderless two-peptide enterocin L50, counterparts of the DdE and DdF proteins exist, and they displayed 79% and 74% of identity to those found in the EntDD14 operon. Moreover, the counterparts of these proteins were found in other enterococci strains, which have homologous EntDD14 clusters, as reported in our previous study [21].

To gain more insights on DdE and DdF, we analyzed in silico their secondary structure and TM domains prediction. This analysis supports the membrane localization of these proteins. Indeed, DdE contains two TM domains, whereas DdF contains six TM domains (Figure 1B), and they seem to have similar structural organization such as YdbS and YdbT proteins of *B. subtilis* (Figure 1A). In addition, the amino acid sequences of DdE and DdF were analyzed using the I-Tasser program that utilizes the resolved 3D structure of proteins deposited in the Protein Data Bank [25,26]. Given the homology between the amino acid sequences and the number and arrangement of TM domains, we assume that the DdE and DdF proteins could contain one and three PH*b*2 domains, respectively. These PH*b*2 domains, which are composed of 72–80 amino acids, are cytosolically oriented. The I-Tasser program predicts the secondary structure in terms of α-helix, β-sheet, and coil. Therefore, the PH*b*2 domains of DdF were shown to be rich in β-sheets (≈58%), while that of DdE contained of ≈41% β-sheet (Table 2).

Furthermore, the I-Taser program has identified several proteins whose 3D structures are significantly similar to those of DdE and DdF. Of note, all these identified proteins are found to be involved in the membrane transport and translocation mechanisms (Table 3). Interestingly, this in silico analysis underpinned a strong homology between the DdE structure and that of the ABC transporter PCAT1 from *Hungateiclostridium thermocellum*. Taken together, these results suggest that the DdE and DdF proteins are membrane proteins that may be involved in the transport of EntDD14.

### 2.2. PH Domain-Containing Proteins DdE and DdF Are Essential for EntDD14 Transport

**Figure 2:**
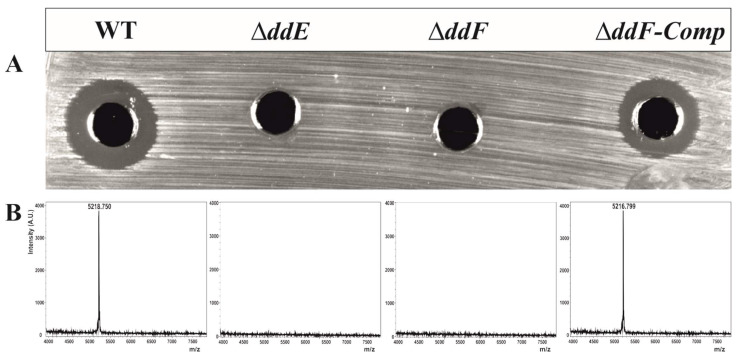
Production of the EntDD14 by *E. faecalis* 14 WT and its derivative mutant strains. (**A**) Agar diffusion test of cell-free supernatants against *L. innocua* ATCC 33090. The inhibition halo indicates DD14 production. (**B**) Detection of the EntDD14 purified from bacterial supernatants by matrix-assisted laser desorption ionization time-of-flight mass spectrometry (MALDI-TOF/MS).

To confirm our in silico analyses, we deleted genes encoding DdE or DdF and analyzed the resulting phenotype of the mutant strains. The deletion of each gene was performed by homologous recombination, using the thermosensitive vector pLT06 [37]. Of note, *E. faecalis* 14 Δ*ddE* and Δ*ddF* mutant strains were obtained, and their genetic backgrounds were confirmed by PCR and sequence analyses. Antibacterial assessment of cell-free supernatant (CFS) from Δ*ddE* or Δ*ddF* mutants was performed by the well-known agar diffusion test (ADT) against *Listeria innocua* ATCC33090 as the bacterial target. Importantly, no inhibitory activity was detected, arguing the absence of EntDD14 in the CFS of the mutant strains (Figure 2A). Therefore, each independently deleted gene entailed the total loss of antimicrobial activity. To confirm this hypothesis, MALDI-TOF/MS analysis was applied on CFS gathered from each mutant strain as well as from the wild type (WT). As expected, EntDD14 was not detected in the CFS from mutant strains (Figure 2B); conversely, that from the WT exhibited a typical peak of 5.2 kDa, corresponding to that of EntDD14, as previously reported by Caly et al. [18].

These independent ways of investigation enabled us to claim that the abolition of DdE or DdF activity impedes transport from the cytoplasm of EntDD14. To strengthen this statement, trans-complementation assays were conducted upon cloning the *ddF* gene into the Gram-positive replicative plasmid pAT18 [38]. The *E. faecalis* ∆*ddF-*complemented strain was generated in the presence of erythromycin. However, the presence of the antibiotic is not compatible with the antimicrobial assay. A study of plasmid stability showed that after 10 and 30 generations without selection pressure, the number of bacteria still harboring the pAT18:*ddF* recombinant vector was 95% and 89%, respectively (data not shown). Thus, we performed all the assays with the complemented strain without erythromycin selection. Following this, the ∆*ddF-*complemented strain was able to secrete again EntDD14, as confirmed by the ADT (Figure 2A) and MALDI-TOF/MS analyses (Figure 2B).

These genetic experimental data showing that *E. faecalis* 14 lacking DdE or DdF protein is clearly unable to transport or translocate out of the cytoplasm EntDD14 bacteriocin reinforce the predictions of the in silico analysis that allocated them a key role in the transport machinery. This surprising result suggests a new pathway in the mode of transport involving PH domain-containing proteins and likely also in the mode of action of the leaderless two-peptide EntDD14.

### 2.3. Loss of DdE or DdF Protein Leads to Overexpression of the EntDD14 Operon

**Figure 3:**
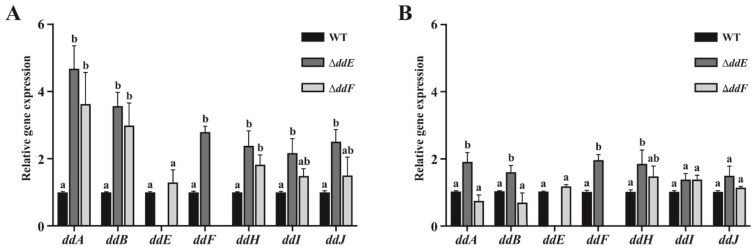
Relative expression values of *ddABEFHIJ* genes in Δ*ddE* and Δ*ddF* mutant strains compared to the WT strain at 5 h (**A**) and 24 h (**B**) of bacterial growth. The *gyrase* gene was used as an internal standard to normalize the values. Statistical significances are represented by letters a and b, which mean *p* < 0.05.

To gain further insight into the EntDD14 mode of transport, a transcriptional analysis was carried out to evaluate the expression of genes involved in the production and transport of EntDD14, primarily those supposed to constitute the ABC transporter (*ddHIJ*). This gene expression experiment was conducted after 5 h (end of logarithmic phase) and 24 h (stationary phase) of growth of the WT strain and its isogenic derivatives Δ*ddE* and Δ*ddF* mutant strains, and the results are shown in Figure 3.

Regarding these results, all the genes tested are constitutively expressed by the WT strain both at the end of the exponential phase and in the stationary phase (24 h). This means that the WT strain is accustomed to tolerating the presence of EntDD14 at a level that does not interfere with its growth or development. In other words, the WT strain must have an intrinsic level of resistance or immunity to its own bacteriocin that may be due to the balance between the production and evacuation of the enterocin, as reflected in the expression of the genes constituting its operon structure.

Indeed, when this expression balance is disrupted by turning off either of the *ddE* or *ddF* genes, the resulting mutants react differently from the WT, and we observe more disturbance at the end of the exponential phase (Figure 3A) than in the stationary phase (Figure 3B), but the changes go in the same direction in the two cases.

At the end of the log phase, *ddA* and *ddB* genes were 4.6- and 3.5-fold overexpressed in the *ΔddE* mutant and 3.6- and 3-fold overexpressed in the *ΔddF* mutant (Figure 3A). For both situations, the overexpression of *ddA* and *ddB* genes suggests that they may be influenced by the *ddE* and *ddF* genes, which could lead, in their corresponding mutants, to an overproduction of EntDD14. Among the genes involved in its extracellular export, mainly *ddF* of the Δ*ddE* mutant is clearly overexpressed (2.7-folds) but at a lower level than for the structural *ddA* and *ddB* genes, which suggests an additional deficit in the ability to evacuate the enterocin outside the cell. As for the other genes of the ABC transporter system (*ddHIJ*), they are overexpressed by a factor of about 2 and mainly in the Δ*ddE* mutant.

This situation occurs also at the stationary phase but with lower overexpression levels and only for the *ΔddE* mutant, since there is even a slight downexpression of *ddAB* genes in Δ*ddF* mutant (Figure 3B), and this may be due to the much-reduced metabolic activity.

**Figure 4:**
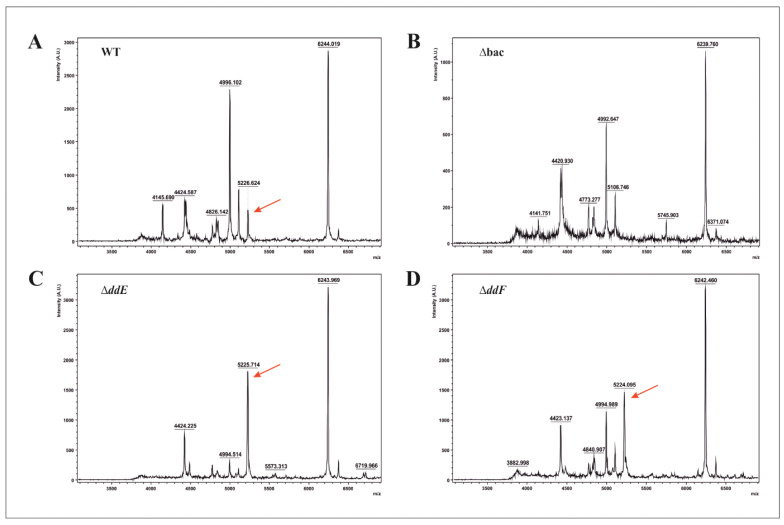
Matrix-assisted laser desorption ionization time-of-flight mass spectrometry (MALDI-TOF/MS) spectra obtained from intracellular fraction proteins of WT (**A**), *Δbac* (**B**), Δ*ddE* (**C**), and Δ*ddF* (**D**) strains. Red arrows mark the peaks corresponding to the EntDD14.

These data indicate overall that (i) *ddAB* genes and (ii) those coding for ABC transporter are expressed in the mutant strains deprived of DdE and DdF proteins, but the cells are unable to externalize EntDD14 outside of the cytoplasm, allowing EntDD14 to accumulate inside the cells. To investigate this point, total intracellular proteins extracted from Δ*ddE* or Δ*ddF* mutant strains were analyzed by MALDI-TOF/MS and compared to those extracted from the WT and the Δ*bac* mutant strain, which were formerly obtained by knocking-out *ddAB* genes and characterized for their inability to produce EntDD14 [21]. In both Δ*ddE* and Δ*ddF* mutant strains, a peak corresponding to EntDD14 with a molecular size of 5.2 kDa was detected (Figure 4C and Figure 4D, respectively). Of note, this peak was also detected in the WT but not in the Δ*bac* mutant strain (Figure 4A and Figure 4B, respectively). Nevertheless, the intensity of the peaks was 400 AU for WT, 1800 AU for Δ*ddE*, and 1600 AU for Δ*ddF*, suggesting an elevated presence of intracellular EntDD14 in the derivative-mutant strains.

As expected, these data confirmed that EntDD14 is more accumulated in Δ*ddE* and Δ*ddF* mutant strains than the WT, arguing that these proteins have a key role in EntDD14 transport out of the cytoplasm.

### 2.4. EntDD14 Accumulated inside the Cytoplasm Induces Cell Toxicity

**Figure 5:**
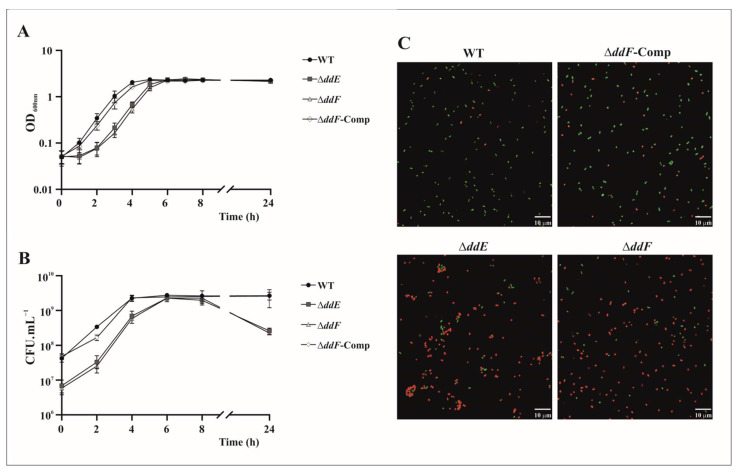
Analysis of bacterial growth of *E. faecalis* 14 strains by measuring the optical density at 600 nm (**A**) and by counting the colony-forming units per mL (**B**). (**C**) Confocal microscope images of bacteria stained with LIVE/DEAD viability kit at 24 h of bacterial growth.

EntDD14 as above-stated accumulates inside the cell when DdE or DdF is missing. To verify the probable deleterious effect of this accumulation, we compared the kinetic growth of the mutants and complemented strains to that of the WT strain (Figure 5). These growth curves revealed discrepancies. The latency phase of the mutant strains is extended in the first hours of growth but reached the same OD_600nm_ than that of the WT strain at the entrance of the stationary phase and remains constant throughout the 24 h of the experiment (Figure 5A). The growth rate (µ) of the mutant strain Δ*ddE* was 1.06 ± 0.06 and that of Δ*ddF* was 1.08 ± 0.07, and it does not differ from that of the WT strain, 1.16 ± 0.05. The complemented Δ*ddF-*Comp strain shows the same behavior as the WT strain with a slightly lower growth rate, 1.01 ± 0.06, which can be ascribed to the presence of the pAT18:*ddF* plasmid. Overall, the mutant strains have registered a loss in cell viability (Figure 5B). The CFU counts indicate that all strains had reached approximately the same number of viable cells at the end of the exponential phase, ≈3 × 10^9^ CFU·mL^−1^. However, after 24 h of growth, Δ*ddE and* Δ*ddF* mutant strains have registered 1 log reduction in CFU·mL^−1^ compared to the WT strain, which represents a 90% loss of cell viability. The loss of cell viability is not necessarily correlated to loss of turbidity of the bacterial culture, as cell lysis seems not to occur.

To confirm this cell-viability feature in the Δ*ddE* and Δ*ddF* mutant strains, we performed a confocal microscopy analysis using the live/dead Bacterial Viability Kit. The ∆*ddF* and ∆*ddE* mutant strains showed similar numbers of bacterial cells but a very low live/dead ratio compared to the WT and the ∆*ddF*-complemented strains (Figure 5C), revealing an abundance of bacteria with compromised membranes that were uncultivable. Therefore, the overall results support that in addition to provoking cell lysis, the intracellular accumulation of EntDD14 is deleterious in the mutant cells lacking *ddE* or *ddF* genes.

## 3. Discussion

The emergence of LLB has opened a new avenue in the field of bacteriocins mainly in understanding their biosynthetic pathway. These bacteriocins, which are composed of one to four peptides [14], do not undergo post-translational modifications or processing, and they are thought to be immediately active after their translation in the cytoplasm. EntDD14 is used here as a model of the two-peptide LLB because of its high sequence homology with its counterparts of the same group.

Bacteriocin transport is most often mediated by Type IV-ABC transporters, which are known to expel toxic molecules out of the producing-cells [5,8,10]. Interestingly, this type of transporter is also involved in the transport of some LLB, such as aureocin A70 [39], aureocin A53 [16], EntD14 [21], lacticin Q, and lacticin Z [40,41]. Nevertheless, the EntDD14 and aureocin A53-producing bacteria altered at least in one protein of the ABC transporters machinery were able to expel only 25% of bacteriocin compared to the WT strain [16,21], arguing the existence of alternative transport pathways.

Here, we propose, for the first time in the history of bacteriocins, a transport role for two PH domain proteins, viz DdE and DdF. These proteins would be dedicated to transport the two-peptide LLB EntDD14 out of the cell. This finding is based on a set of complementary data including in silico analyses, genetic experimental evidence, and RT-qPCR tools as well as mass spectrometry and microscopy. The mutant strains ∆*ddE* and ∆*ddF*, lacking DdE and DdF, resulted in a loss of EntDD14 transport, albeit genes (*ddABHIJ*) coding for EntDD14 and the ABC transporter were expressed. Consequently, the bacteriocin remained trapped inside the cell, leading finally to toxic internal activity. These data showed that LLB are indeed active inside the cytoplasm.

The deleterious effect of an LLB was also reported for the lacticin Q when its coding gene was expressed in the absence of the *lnqBCDEF* genes [40]. Lacticin Q is a single-peptide LLB produced by *Lactococcus lactis* QU 5. The deletion of any of the *lnqCDEF* genes abolished the bacteriocin production. These authors suggested that *lnqCD* along with *lnqB* genes could play a role of accessory proteins to the ABC transporter formed by *lnqEF* genes [41]. It has been also reported that deletion of the *orf8* gene in *S. aureus* A53 that produces another single-peptide LLB aureocin A53, enabled bacteriocin production [16]. The genetic determinants required for aureocin A53 synthesis are organized in at least four transcriptional units encoded by the 10.4-kb plasmid pRJ9. One of these units corresponds to the *orf7* and *orf8* genes. Interestingly, the sequence analyses of Orf7 and Orf8 from *S. aureus* A53 and LnqC and LnqD from *L. lactis* QU 5 showed homology to *B. subtilis* YdbS and YdbT proteins, respectively (Table 1), and also a certain level of homology with DdE and DdF proteins from *E. faecalis* 14.

The presence of proteins DdE and DdF with such domains were as well encountered in different operons related to two-peptide LLB L50 produced by different *E. faecium* strains. In all of them, these PH domain-containing proteins are highly conserved, reaching a homology score of 74% for DdE and 78% for DdF. These findings showed that this new transport pathway based on PH domain-containing proteins is not unique to EntDD14 but is common to several single- and two-peptide LLB.

The in silico analysis of DdE and DdF proteins enabled establishing the preliminary snapshot of their structures. Thus, DdE has structural similarities to proteins exerting electron transfer activity across the cell membrane. However, modeling of DdE did not explain how its PH*b*2 domain can interact in a transport function. On the other hand, the DdF protein has partial structural homology to ABC transporter proteins (Table 3), which are composed of two differentiated domains. The first is a dimeric cytoplasmic nucleotide-binding domain (NBD), and the second is a homo- or heterodimeric transmembrane domain (TMD). Of note, DdF was in turn predicted with six transmembrane α-helices, which concurs with the consensus organization of TMD, which most often includes two sets of six hydrophobic helices [5,8]. The highest structural homology of DdF was obtained with the TMD and the C39 protease domain of the PCAT1 transporter from *H. thermocellum* [31]. This transporter belongs to the SunT-type ABC transporters, and it cleaves the leader peptide prior to its translocation across the membrane [5], where the C39 domain is responsible for the specific recognition of the peptide and its cleavage. The putative PH*b*2 domains in DdF seem to conform under a similar structure to that of the C39 domain, which could specifically recognize EntDD14 and facilitates its transport across the membrane.

The PH domain is widely encountered in Eukaryotic proteins where their sequences are not necessarily conserved. Nevertheless, all these proteins present the same folding in their structure, named the PH superfold, which is also shared with other domains such as the phosphotyrosine binding (PTB) domain, Enabled/VASP homology (EVH1) domain, and Ran-binding domain (RanBD). For this reason, the PH superfold was suggested as a scaffold for multiple functions [42]. The PH domain superfold includes proteins involved in binding to a variety of phosphoinositide and protein ligands, which mediate protein targeting to the membrane and protein interactions in signal transmission processes [43].

The PH*b*2 domain was described in exig_2160 protein from *Exiguobacterium sibiricum* 255-15. This protein is uncharacterized, and its function remains unknown, but its structural study showed the oligomerization of the protein involving twelve monomers binding by the PH*b*2 domain [24]. The PH*b*2 domains in exig_2160 protein are composed by seven β-sheets and a C-terminal α-helix. The prediction of DdE and DdF PH*b*2 domains’ secondary structure showed also a predominance in β-sheet. Thus, we believe that the putative PH*b*2 domains in DdE and DdF proteins may be involved in their own interactions and/or in the recognition of EntDD14, leading to its transport across the membrane.

Here, we firmly believe that the DdE and DdF proteins, as well as their counterparts in the bacteriocin-producing bacteria, are not acting as accessory proteins as previously reported by Iwatini et al. [41] but constitute new carriers dedicated for LLB. Both DdE and DdF are simultaneously required for two-peptide LLB EntDD14 transport. Moreover, in all the YdbS/YdbT-like couple proteins reported in Table 1, the genes were not found associated with ABC transporter genes, which strengthens our data and thus allocates a transport function to DdE and DdF. Further analyses aimed to understand interactions between DdE and DdF as well as interactions between DdE and EntDD14 or DdF and EntDD14 constitute our next focus.

## 4. Materials and Methods

### 4.1. Bacterial Strains and Growth Conditions

Bacteria used in this work are listed in Table 4. *Enterococcus faecalis* strains were routinely grown in M17 medium supplemented with 0.5% glucose (GM17), at 37 °C. *Escherichia coli* strains were grown in Luria–Bertani (LB) broth at 37 °C by shaking at 160 rpm. *Listeria innocua* ATCC33090 strain was grown in Brain–Heart Infusion (BHI) broth at 37 °C. When bacteria carried the pLT06 plasmid or its derivatives, the medium was supplemented with chloramphenicol (Cm) at 10 µg·mL^−1^ for *E. coli* and at 15 µg·mL^−1^ for *E. faecalis*. When bacteria carried the pAT18 plasmid or its derivatives, the medium was supplemented with erythromycin (Em) at 150 µg·mL^−1^ for *E. coli* and *E. faecalis*.

### 4.2. Construction of the ΔddE and ΔddF Strains

The ddE and ddF genes were separately deleted from the E. faecalis 14 chromosome by recombinant exchange with the surrounding regions of the genes, using a strategy based on the conditional replication of the pLT06 plasmid [37]. The oligonucleotides used for this purpose are listed in Table 5. The flanking regions of each gene were amplified by PCR using the genomic DNA of E. faecalis 14 as the template, the corresponding 1F/2R (upstream) and 3F/4R (downstream) oligonucleotide pairs, and the Phusion^TM^ High-Fidelity DNA Polymerase Mix (ThermoFisher Scientific, Waltham, MA, USA). The 2R and 3F oligonucleotides have a region of 24 complementary nucleotides, which allowed us to amplify both fragments together, using a mixture of the two fragments as a template and the 1F/4R oligonucleotide pairs. This complementary region is the one that replaces the deleted gene in the mutant strain. Thus, four stop codons were inserted to avoid any undesired translation. Final amplicons were purified from 1% agarose gel. They as well as the pLT06 plasmid were digested with PstI and NcoI restriction enzymes (ThermoFisher Scientific, USA) and finally ligated overnight with T4 ligase (ThermoFisher Scientific, USA). The ligation mixtures were used to transform E. coli XL1-Blue by heat shock. Transformed colonies were selected on LB agar plates supplemented with Cm at 10 µg·mL-1 and X-gal at 80 µg·mL-1. A blue positive colony of each construction was cultured to extract the pLT06:ΔddE and pLT06:ΔddF plasmids. These plasmids were confirmed by PCR and sequencing. A quantity of 0.5 µg of both plasmids was used to transform 50 µL of electro-competent cells of E. faecalis 14 by electroporation (25 µF, 2.5 kV, and 200 Ω in pre-chilled 0.2 cm cuvettes). Transformed colonies were selected on M17-agar plates supplemented with Cm at 15 µg·mL-1 and X-gal at 80 µg·mL-1 at 30 °C.

The first recombinant event was induced as follows. Transformant strains harboring pLT06:Δ*ddE* or pLT06:Δ*ddF* were grown in GM17 broth supplemented with Cm at 15 µg·mL-1 at 30 °C for 2 h; then, they were shifted to 42 °C for 4 h. At this temperature, the replicon of the pLT06 plasmid was disabled, and with the selection pressure of the Cm, its integration into the chromosome by recombination was forced. Larger blue colonies grown on M17-agar plates (Cm+X-gal) were verified by PCR using the outer 5F oligonucleotides with one of the oligonucleotides located on the plasmid (OriF or KS05seqR, in Table 5). One of these colonies from each mutant was subjected to the second recombinant event. The colonies were grown in GM17 broth in the absence of Cm at 30 °C. The cultures were diluted several times growing until stationary phase. At 30 °C, the replicon of pLT06 is functional, provoking the excision from the chromosome, and without the antibiotic pressure, the loss of the plasmid was favored. White colonies grown in M17-agar-X-gal plates without Cm were screened for the mutant genotype by PCR using the outer 5F/6R oligonucleotide pairs. The suitable mutants for *ddE* and *ddF* gene deletions were verified by sequencing the surrounding genetic environment.

### 4.3. Complementation of the E. faecalis ΔddF Mutant Strain

To complement the Δ*ddF* mutant strain, the gene *ddF* was cloned into the pAT18 plasmid [38]. The DNA fragment containing the *ddF* gene was amplified by PCR using the ddF Comp oligonucleotides (Table 5). The amplicon and the pAT18 plasmid were digested with KpnI and BamHI restriction enzymes and ligated overnight with the T4 ligase. The ligation mixture was used to transform *E. coli* XL1-Blue by heat shock. Transformed colonies were selected on LB agar plates supplemented with Em at 150 µg·mL^−1^ and X-gal at 80 µg·mL^−1^. A white positive colony of each construction was cultured to extract the pAT18:*ddF* plasmid. This plasmid was confirmed by PCR and sequencing. Then, 0.5 µg of the plasmid was used to transform 50 µL of electro-competent cells of *E. faecalis* 14 Δ*ddF* strain by electroporation (25 µF, 2.5 kV, and 200 Ω in pre-chilled 0.2 cm cuvettes). Transformed colonies were selected on M17 agar plates supplemented with Em at 150 µg·mL^−1^ and X-gal at 80 µg·mL^−1^.

To test the antibacterial activity of the complemented strain, the Em of the medium must be removed. Nonetheless, we performed a plasmid stability study to analyze the presence of the plasmid over time. Thus, the complemented strain was cultured during 10 and 30 generations in GM17 without Em. At these points, the percentage of cells harboring the plasmid was calculated by counting the UFC·mL^−1^ on M17 agar plates containing or not Em at 150 µg·mL^−1^.

### 4.4. Antimicrobial Activity against L. innocua

The screening of anti-*L. innocua* activity of the cell-free supernatant from *E. faecalis* 14 WT and mutant strains, as well as purified EntDD14 was performed using the well-known agar diffusion method [18]. Briefly, a uniform layer of *Listeria* culture was deposited on a soft BHI-agar (1%) plate using a swab. Then, wells of 5 mm diameter were aseptically made in the agar. After that, 50 μL of cell-free supernatants and/or purified EntDD14 were introduced, separately, into the wells. Then, the plates were incubated at 4 °C for 1 h and then overnight at 37 °C. The absence or presence of inhibitory zones around the wells was recorded.

### 4.5. RNA Isolation and RT-qPCR

Quantitative reverse transcription PCR (RT-qPCR) analysis was carried out to study the expression of genes involved in enterocin DD14 production and transport in *E. faecalis* 14 WT and Δ*ddE* and Δ*ddF* strains. At 5 h and 24 h, cultures of these strains were performed. Cells were harvested by centrifugation (10,000× *g* during 10 min at 4 °C), and total RNA was extracted using the NucleoSpin^TM^ RNA Plus columns (Macherey-Nagel, Hoerdt, France). The quantity and quality of RNA samples were determined by capillary electrophoresis, using an Agilent 2100 Bioanalyzer (Agilent Technologies, Les Ulis, France), and a minimal RNA integrity number (RIN) of 8 was required for all samples. First, 1 µg of total RNA from each sample was treated with DNase (Thermo Fisher Scientific) to remove all traces of DNA. After DNase inactivation with EDTA (Thermo Fisher Scientific), the RNA was converted to complementary DNA (cDNA) using the RevertAid H Minus First Strand cDNA Synthesis Kit (Thermo Fisher Scientific). Changes in the mRNA expression of several genes (*ddA*, *ddB*, *ddE*, *ddF*, *ddH*, *ddI*, and *ddJ*) were monitored by real-time qPCR, performed with the Brilliant III SYBR Green QPCR Master Mix (Agilent Technologies) on a “CFX Connect Real-Time PCR Detection System” thermocycler (BIO-RAD). The oligonucleotides used are listed in Table 5. The mean CT of each sample was normalized against the housekeeping gene (*gyrase*) and the corresponding control. The relative quantification of each gene was calculated by the 2^−(ΔΔCt)^ method, using the Bio-Rad’s CFX Manager software.

### 4.6. Intracellular Protein Extraction

Overnight cultures of *E. faecalis* 14 strains (WT, *Δbac*, Δ*ddE*, Δ*ddF*, and Δ*ddF*-Comp) were diluted in fresh GM17 medium and grown at 37 °C for 24 h. Then, the cells were harvested by centrifugation (10,000× *g* during 10 min at 4 °C) and resuspended in lysis buffer (20 mM Tris-HCl pH 8.0, 300 mM NaCl) and then sonicated in an ice bath using the OmniRuptor 4000 Ultrasonic Homogenizer (OMNI International, Kennesaw, GA, USA). Their concentrations were determined by the bicinchoninic acid (BCA) assay protein kit (Sigma-Aldrich, St Louis, MI, USA), following the manufacturer’s recommendations.

### 4.7. Purification of the Leaderless Two-Peptides EntDD14

EntDD14 was purified from the supernatant of *E. faecalis* 14 WT, Δ*ddE*, Δ*ddF*, and Δ*ddF*-Comp strains. The purification procedure was adapted from Abriouel et al. [44] as follows. Each strain was grown in 200 mL of GM17 at 37 °C for 24 h. After harvesting of the cultures by centrifugation (10,000× *g* during 10 min at 4 °C), the obtained cell-free supernatants were incubated at room temperature for 24 h with the CM Sephadex^®^ C-25 resin (GE Healthcare Life Sciences, Issaquah, WA) with shaking at 90 rpm. The mixture was poured into a chromatography column, where the resin was allowed to settle. Then, the resin was washed with 400 mL of distilled water and 200 mL of 0.5 M NaCl. Then, the resin-bound DD14 was eluted with 30 mL of 1.5 M NaCl. The solution containing DD14 was desalted by gel filtration using PD MidiTrap G-10 columns (GE), eluting with milliQ water. The pure EntDD14 was quantified using the BCA assay protein kit (Sigma-Aldrich) and then, it was dried in aliquots by miVac Sample Concentrators (SP Scientific, Gardiner, NY, USA) for its storage. When used, an aliquot of pure DD14 was resuspended in the appropriate volume of MilliQ water to achieve the desired concentration.

### 4.8. Detection of EntDD14 by MALDI-TOF/MS

EntDD14 in cell-free supernatants as well as in intracellular protein fractions of WT and mutant strains were detected by matrix-assisted laser desorption ionization time-of-flight mass spectrometry (MALDI-TOF/MS). The analysis was carried out using the Autoflex Speed MALDI TOF/TOF equipment (Bruker Daltonics, Bremen, Germany), and spectra were obtained using flexAnalysis software (Bruker Daltonics, Germany). When required, the samples were concentrated using Pierce^TM^ C18 tips (Thermo Scientific).

### 4.9. Evaluation of the Effect on the Producer Strains by Ent DD14 Intracellular Accumulation

To evaluate the effect of accumulated EntDD14 inside the cells in mutant strains, we examined the bacterial growth of WT, Δ*ddE*, Δ*ddF*, and Δ*ddF*-Comp strains. Overnight cultures were diluted in fresh GM17 medium to OD_600nm_ = 0.05, and the bacterial growth was followed by hourly measuring the OD_600nm_ with a spectrophotometer (Aqualabo, Champigny sur Marne, France) during 24 h. The UFC·mL^−1^ were obtained by counting on M17 agar plates at 0, 2, 4, 6, 8, and 24 h of growth.

### 4.10. Confocal Laser Scanning Microscopy

Cultures of WT, Δbac, Δ*ddE*, Δ*ddF*, and Δ*ddF*-Comp strains were treated with the LIVE/DEAD™ *Bac*Light™ Bacterial Viability Kit (Thermo Fisher Scientific, Landsmeer Netherlands) to analyze the viability of the bacteria at 24 h of growth. The staining procedure was carried out following the manufacturer’s instructions. Stained bacterial solutions were imaged with a ZEISS LSM 780 confocal laser scanning microscope equipped with a 40x/1.3 oil immersion objective (Carl Zeiss Micro Imaging GmbH, München, Germany). The SYTO 9 dye was excited with a laser at 488 nm and detected between 493 and 560 nm; and the propidium iodide dye was excited at 561 nm and detected between 584 and 718 nm. The images were acquired with the Zen software (Carl Zeiss Micro Imaging GmbH, Germany), and analyzed with the ImageJ software (National Institute of Health, Bethesda, MD, USA).

### 4.11. Bioinformatics

The *E. faecalis* 14 genome sequence was obtained from GenBank under the accession number CP021161.1. The sequences were analyzed using the Basic Local Alignment Search Tool of the National Center for Biotechnology Information (BLAST-NCBI: https://blast.ncbi.nlm.nih.gov/Blast.cgi, accessed on 2 April 2020) and the SnapGene^®^ 4.3.7 tool. The prediction of transmembrane helices in proteins was carried out with the TMHMM server v. 2.0 (http://www.cbs.dtu.dk/services/TMHMM-2.0/ accessed on 14 June 2020) [45]. The structural model predictions were obtained using the online server Iterative Threading ASSEmbly Refinement (I-Tasser: https://zhanglab.ccmb.med.umich.edu/I-TASSER/ accessed on 4 May 2020) [25,26].

### 4.12. Statistical Analysis

All the results presented in this work were obtained from three independent experiments, and the data are expressed as the mean standard deviation. RT-qPCR results were statistically analyzed by one-way analysis of variance (ANOVA), followed by the Tukey’s test to determine the significant differences between the variables at *p* values < 0.05.

## Figures and Tables

**Table 1:** Members of the bacterial proteins families YdbS and YdbT containing PH*b*2 domains from *Bacillus subtilis* ssp. *subtilis* 168.

Protein	Size ^a^	Accession No	% I ^b^	% P ^c^	Bacteria	Accession No
YdbS ^d^	159	NP_388340.1	100	100	*Bacillus subtilis* ssp. *subtilis* 168	NC_000964.3
YdbS	159	VEH76774.1	68	79	*Bacillus licheniformis* NCTC10341	LR134392.1
Lin0881	160	WP_003761286.1	40	58	*Listeria innocua* Clip11262	NC_003212.1
SA1878	159	WP_001287087.1	23	43	*Staphylococcus aureus* ssp. *aureus* N315	NC_002745.2
NCgl0612	149	WP_003860754.1	24	47	*Corynebacterium glutamicum* ATCC 13032	NC_003450.3
MT_RS06490	177	WP_003406264.1	25	48	*Mycobacterium tuberculosis* CDC1551	NC_002755.2
DdE	141	-	17	47	*Enterococcus faecalis* 14	CP021161.1
YdbT ^d^	493	NP_388341.1	100	100	*Bacillus subtilis* ssp. *subtilis* 168	NC_000964.3
YdbT	493	VEH76775.1	52	72	*Bacillus licheniformis* NCTC10341	LR134392.1
Lin0882	494	WP_010990663.1	31	51	*Listeria innocua* Clip11262	NC_003212.1
SA1877	527	WP_001294626.1	23	46	*Staphylococcus aureus* ssp. *aureus* N315	NC_002745.2
NCgl0613	471	WP_011013786.1	17	45	*Corynebacterium glutamicum* ATCC 13032	NC_003450.3
MT_RS06485	487	WP_003898781.1	17	38	*Mycobacterium tuberculosis* CDC1551	NC_002755.2
DdF	458	-	18	44	*Enterococcus faecalis* 14	CP021161.1

^a^ The protein size is given in number of amino acid residues. ^b^ Percentage of identical homology compared with the template *B. subtilis* 168 YdbS or YdbT proteins. ^c^ Percentage of positive homology compared with the template *B. subtilis* 168 YdbS or YdbT proteins. ^d^ Model proteins used as template for the homology analysis.

**Table 2:** Prediction of the secondary structure of DdE and DdF PH*b*2 domains.

Protein	PH*b*2 Domain	Size ^a^	% β-Sheet	% α-Helix	% Coil
DdE	67–138	72	41.67	43.05	15.28
DdF	54–133	80	57.50	13.75	28.75
	222–295	74	58.11	13.51	28.38
	382–453	72	58.33	25.00	16.67

^a^ The protein size is given in number of amino acid residues.

**Table 3:** Proteins structurally close to DdE and DdF obtained with the I-TASSER program.

PDB	TM-Score ^a^	Characteristic	Role	Organism	Reference
DdE structurally close proteins					
6f0k	0.538	Respiratory alternative complex III	Electron transfer membrane protein	*Rhodothermus marinus* DSM 4252	[27]
6btm	0.533	Respiratory alternative complex III	Electron transfer membrane protein	*Flavobacterium johnsoniae* UW101	[28]
3e0s	0.521	Uncharacterized protein	Structural genomics/unknown function	*Chlorobaculum tepidum*	unpublished
3jrt	0.518	Integron cassette protein Vpc_cass2	Structural genomics/unknown function	*Vibrio paracholerae*	[29]
7d3e	0.511	DUOX1-DUOXA1 in low-calcium state	Electron transport	Homo sapiens	unpublished
5u6o	0.507	HCN1 hyperpolarization-activated cyclic nucleotide-gated ion channel	Transport protein	Homo sapiens	[30]
DdF structurally close proteins					
4ry2	0.712	Peptidase-containing ABC transporter PCAT1	Transport protein/hydrolase	*Hungateiclostridium thermocellum* ATCC 27405	[31]
3qf4	0.434	Heterodimeric ABC transporter	Transport protein	*Thermotoga maritima*	[32]
4mrn	0.433	Bacterial Atm1-family ABC transporter	Transport protein	*Novosphingobium aromaticivorans* DSM 12444	[33]
3ffz	0.423	Domain organization in butulinum neurotoxin type E	Hydrolase/translocation	*Clostridium botulinum*	[34]
6tqe	0.413	ABC transporter Rv1819c	Transport protein	*Mycobacterium tuberculosis*	[35]
5mkk1	0.407	Heterodimeric ABC transporter TmrAB	Transport protein	*Thermus thermophilus* HB27	[36]

^a^ TM score assesses the topological similarity of protein structures. Values are between 0 and 1. A score higher than 0.5 indicates generally the same fold in SCOP/CATH.

**Table 4:** List of the bacteria used in this work.

Bacteria	Plasmids	Resistance	Characteristics	Reference
*Escherichia coli*				
XL1-Blue	–	–	Plasmid-free type strain used for plasmid cloning	Agilent Technologies
XL1-Blue [plT06]	pLT06	Cm ^R^	Source of the pLT06 plasmid used for mutant strategies	[21]
XL1-Blue [pLT06:Δ*ddE*]	pLT06:Δ*ddE*	Cm ^R^	Derivative of pLT06 by cloning of a 2219 pb DNA fragment harboring flanked regions of *ddE* gene	This study
XL1-Blue [pLT06:Δ*ddF*]	pLT06:Δ*ddF*	Cm ^R^	Derivative of pLT06 by cloning of a 2019 pb DNA fragment harboring flanked regions of *ddF* gene	This study
XL1-Blue[pAT18]	pAT18	Em ^R^	Source of pAT18 used for complementation studies, based on the inducible *lac* promoter	[21]
XL1-Blue [pAT18:*ddF*]	pAT18:*ddF*	Em ^R^	Derivative of pAT18 by cloning of *ddF* gene under the control of *lac* promoter	This study
*Enterococcus faecalis*				
14	–	–	Natural strain isolated from meconium	[17]
14 Δ*ddE*	–	–	Deletion mutant strain of *ddE* gene	This study
14 Δ*ddF*	–	–	Deletion mutant strain of *ddF* gene	This study
14 Δ*ddF*-Comp	pAT18:*ddF*	Em ^R^	Derivative of pAT18 by cloning of *ddF* gene under the control of *lac* promoter	This study
*Listeria innocua*				
ATCC33090	–	–		[21]

^R^: resistant.

**Table 5:** List of oligonucleotides used in this work.

Oligonucleotide	Sequence 3′-5′	Utilization	Amplicon Size (pb)
ddE 1F-PstI	ATTAAACTGCAGTGATATACAATTTATATGAACAA	Amplification of *ddE* upstream fragment	1136
ddE 2R-Stop	CATTCACTAGGATCCTTAGACTTATACAAATTCATTTTTCATTGAA		
ddE 3F-Stop	TAAGTCTAAGGATCCTAGTGAATGAAGAAGAGGTTATTAGATGAA	Amplification of *ddE* downstream fragment	1107
ddE 4R-NcoI	ATTAAACCATGGTATCTATAGCCATAAAAATAGCC		
ddE 5F	AGATATATTGATATACAATTTATATG	Outer primer; verification of the plasmid integration	–
ddE 6R	ACTATCAAAATATCTCTTACATAC		
ddF 1F-PstI	ATTAAACTGCAGGTCTATTATAGGAGGTAAAAATG	Amplification of *ddF* upstream fragment	1016
ddF 2R-Stop	CATTCACTAGGATCCTTAGACTTATTTCATCTAATAACCTCTTCTTTTA		
ddF 3F-Stop	TAAGTCTAAGGATCCTAGTGAATGTCGTAGGAGGATAGAATGAAC	Amplification of *ddF* downstream fragment	1027
ddF 4R-NcoI	ATTAAACCATGGGGCTTTTTTCATTTCATCATCC		
ddF 5F	AAACGAAAGGGGACTGTAGC	Outer primer; verification of the plasmid integration	–
ddF 6R	TCAATTTTATTATCAGCTTCAGC		
ddF CompF-KpnI	AAAAGGTACCAATAAAAGAAGAGGTTATTAGATG	Cloning of the *ddF* gene in pAT18	1434
ddF CompR-BamHI	AAAAGGATCCTGTTCATTCTATCCTCCTACG		
oriF	CAATAATCGCATCCGATTGCA	Cloning verification in pLT06 plasmid	–
Ks05R	CCTATTATACCATATTTTGGAC		
PU	GTAAAACGACGGCCAGT	Cloning verification in pAT18 plasmid	–
PR	CAGGAAACAGCTATGAC		
EntAL	ATGGGAGCAATCGCAAAAT	Amplification of internal *ddA* gene fragment for qPCR	100
EntAR	TAATTGCCCATCCTTCTCCA		
EntBL	AAAGTTTGGATGGCCATTTATT	Amplification of internal *ddB* gene fragment for qPCR	106
EntBR	TCAATGTCTTTTTAACCATTTTTCA		
EL	ACAAGAACATATACATTTGTGAAGGA	Amplification of internal *ddE* gene fragment for qPCR	95
ER	AACATATTCTGTTTCAATTACCGTGT		
FL	AGGAAAATGTTGATTTGGTGTTT	Amplification of internal *ddF* gene fragment for qPCR	100
FR	TCCAATGAAGATAACAAGACAAAAA		
HL	TGGTCAAGAAATCAATGAAAATG	Amplification of internal *ddH* gene fragment for qPCR	89
HR	CTAGAGATTGGGTTTGTTCTTCC		
IL	GGGATTTATCGATCGTAAGTTTG	Amplification of internal *ddI* gene fragment for qPCR	86
IR	TTTTAGAAAGAATGTCATCTGCTGT		
JL	AGAAGGAGTTAAACCCGATAAGG	Amplification of internal *ddJ* gene fragment for qPCR	87
JR	TCATATTCTCCCAGATGTCTCAA		

## Data Availability

Not applicable.
